# Behavioral and Brain Gene and Protein Changes in Female Mice Consuming Ethanol During Pregnancy and Lactation

**DOI:** 10.3390/biom15091239

**Published:** 2025-08-27

**Authors:** Daniela Navarro, Francisco Navarrete, Nerina Villalba, Abraham B. Torregrosa, Laura Caltana, Ani Gasparyan, Alicia Brusco, Jorge Manzanares

**Affiliations:** 1 Instituto de Neurociencias, Universidad Miguel Hernández-CSIC, Avda. de Ramón y Cajal s/n, San Juan de Alicante, 03550 Alicante, Spain; dnavarro@umh.es (D.N.); fnavarrete@umh.es (F.N.); a.bailen@umh.es (A.B.T.); agasparyan@umh.es (A.G.); 2 Redes de Investigación Cooperativa Orientada a Resultados en Salud (RICORS), Red de Investigación en Atención Primaria de Adicciones (RIAPAd), Instituto de Salud Carlos III, MICINN and FEDER, 28029 Madrid, Spain; 3 Instituto de Investigación Sanitaria y Biomédica de Alicante (ISABIAL), 03010 Alicante, Spain; 4 Instituto de Biología Celular y Neurociencia “Prof. E. De Robertis” (IBCN), Universidad de Buenos Aires-CONICET, Buenos Aires C1121ABG, Argentina; nvillalba@fmed.uba.ar (N.V.); lcaltana@fmed.uba.ar (L.C.); aliciabrusco@gmail.com (A.B.)

**Keywords:** ethanol consumption, pregnancy, gestation, behavioral alteration, molecular changes

## Abstract

Alcohol consumption during pregnancy and lactation remains a significant global issue. Preventive policies have proven insufficient, and consumption rates remain high, mainly due to a lack of awareness, the misconception that only high alcohol intake harms the fetus, inconsistent medical advice, and pre-existing alcohol use disorders. Alcohol consumption is linked to child harm during critical stages of development. Using a recently published model of fetal alcohol spectrum disorder (FASD) developed by our group, we analyzed brain changes in mothers who consumed alcohol during pregnancy and lactation and the behavioral consequences at the emotional-cognitive level and in caring for their offspring. We also considered the previous drinking history, using the paradigm of voluntary pre-gestational alcohol consumption. In addition, from gestational day 7 (GD7) until the day of sacrifice, mothers received a 3 g/kg dose of alcohol every 12 h via gavage. Our findings revealed deficiencies in maternal care, anxiety and depressive-like behavior, and aversive stimulus learning disturbances. These were associated with changes in gene targets linked to stress-axis regulation, reward circuits, and neuroplasticity. Additionally, we observed increased microgliosis and astrogliosis, indicating neuroinflammation in brain regions involved in cognition and emotional states’ regulation.

## 1. Introduction

The adverse effects of alcohol consumption during gestation and lactation remain a significant public health problem worldwide Broccia, Munch [1,2,3]. A recent retrospective cohort study found that of 1,432,979 newborns in the US, 2.1% of infants had injuries related to maltreatment, and 3.1% of infants had morbidities associated with alcohol use during pregnancy. Notably, most US pregnancy-specific alcohol policies are not related to reductions in childhood injury or morbidity [4]. On the other hand, the global prevalence of fetal alcohol spectrum disorder (FASD) among children and adolescents is 7.7 per 1000 people, and the WHO European Region has the highest prevalence [5].

Alcohol consumption during pregnancy and lactation may be due to several factors, such as the belief that only a large quantity of alcohol has adverse effects on the fetus, unclear advice from medical practitioners, and a previous history of alcohol use disorders (AUD) [6]. Treatment of AUD in pregnant and breastfeeding mothers is critical, as several factors need to be considered, including relapse prevention, management of withdrawal symptoms during pregnancy, and prevention of drinking while breastfeeding [7]. Thus, perinatal alcohol consumption continues to be a key public health problem.

Maternal alcohol use disorder is associated with poor parenting skills, inadequate supervision, family conflict, and an increased risk of child abuse and neglect [8]. Several studies in humans and rats have shown that chronic alcohol exposure can produce cognitive impairment. In humans, alcohol exposure produces progressive cognitive dysfunction, loss of neural plasticity, reduced GABAergic inhibition, and increased glutamatergic excitation, joined with impulsivity and cognitive impairment consisting of attentional and verbal memory deficits [9,10,11]. In addition, women with AUD are more likely than men to have comorbid disorders such as anxiety and depression [12]. Women also metabolize alcohol differently than men, reaching higher and longer-lasting blood alcohol concentrations. This fact may contribute to the development of more serious problems and neurotoxicity at lower levels of consumption [13,14]. In rats, chronic exposure produces cognitive and motor function impairment [15,16,17,18,19]. However, no animal models are showing changes in offspring care, anxiety, depressive-like behavior disturbances, and maternal brain alterations produced by alcohol consumption during gestation and lactation. Knowledge of maternal behaviors related to the care of offspring and their emotional and cognitive state is of fundamental importance to better understand possible alterations in offspring due to these factors.

Recently, we published a new murine model of FASD using a perinatal alcohol exposure (PAE) paradigm by ethanol gavage administration (3 g/kg/12 h, p.o.) from GD 7 to pregnant females with a previous alcohol consumption history (voluntary ethanol consumption), where the alteration produced by alcohol consumption during gestation and lactation only in the offspring was observed [20]. Once consumption and preference for 10% ethanol stabilized, only females with high consumption and ethanol preference were selected. They were then mated with male mice. Once pregnancy was confirmed, starting on GD7, throughout all behavioral tests and until a few hours before sacrifice, mothers previously exposed to voluntary ethanol consumption were administered 3 g/kg of ethanol (p.o., twice daily, every 12 h). In the present study, using this model of FASD, we evaluated the effects of alcohol consumption on the maternal care of the offspring, as well as the behavioral and neurobiological alterations in mothers. We found notable issues in several maternal care traits, together with anxiety, depression-like, and cognitive (aversive stimulus learning) alterations. Furthermore, changes in the gene expression of targets involved in regulating stress response, reward, and neuroplasticity were obtained. In addition, there was an increase in microgliosis and astrogliosis, evidencing inflammatory processes in brain areas of the limbic system involved in cognition and emotional state regulation, such as the hippocampus (HIPP), insula, amygdala (Amy), and cingulate cortex.

This study aims to underscore that alcohol consumption during gestation and lactation can lead to maternal alterations in caregiving and behavior, which may indirectly contribute to neurodevelopmental disturbances in the offspring.

## 2. Materials and Methods

### 2.1. Animals

We used 56 C57BL/6 J male and female mice in all experiments. Thirty-six female and twenty male 5-week-old mice were purchased from Charles River Laboratories (Lille, France). After their arrival, mice were individualized and left undisturbed for one week to acclimate to the animal housing room. Subsequently, females were exposed to a voluntary ethanol consumption paradigm before breeding began. All animals were maintained in the Animal Facilities Service of the Universidad Miguel Hernández (SEA-UMH) under controlled environmental conditions of temperature (23 ± 2 °C), humidity (60 ± 10%), and a 12 h light-dark cycle (lights on from 08:00–20:00 h). All experimental procedures complied with the Spanish Royal Decree 53/2013, the Spanish Law 32/2007, and the European Union Directive of the 22nd of September 2010 (2010/63/UE) regulating the care of experimental animals and were approved by the Ethics Committee of Miguel Hernández University. Animal studies are reported in compliance with the ARRIVE guidelines [21,22].

### 2.2. Perinatal Alcohol Exposure Procedure

**Figure 1:**
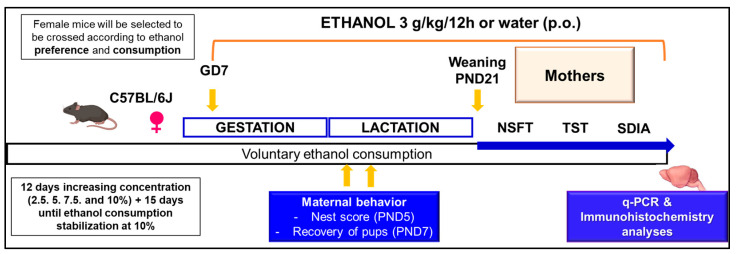
Summary of the experimental procedures. Female mice were exposed to a voluntary ethanol consumption paradigm, achieving a stable 10% ethanol solution consumption and preference before crossing. At GD7, ethanol gavage was started at a dose of 3 g/kg/12 h (p.o.) until the sacrifice. After the 1st week of weaning, behavioral evaluations were initiated to determine anxiety and depressive symptoms and aversive learning alterations using different experimental paradigms. At the end of these behavioral studies, mice were sacrificed and frozen, or perfused brain samples were obtained to perform the proposed molecular studies. GD: gestational day, p.o.: oral administration. TST: tail suspension test, NSFT: novelty suppressed feeding test, SDIA: step-down inhibitory avoidance.

Perinatal exposure of female mice to ethanol was based on our published method [20]. Female C57BL/6J mice were exposed to voluntary ethanol consumption in a two-bottle choice paradigm from PND 42. Female mice were housed individually in cages with two water bottles to acclimate and avoid potential stress. Animals were then divided into two groups: one exposed to two bottles, both containing only tap water (*n* = 16), and another exposed to two bottles, one always containing tap water and the other containing increasing concentrations of ethanol (2.5, 5, 7.5, and 10 percent ethanol, 3 days for each concentration) (*n* = 20). Bottles were alternated to prevent any place preference bias. Food was available ad libitum, and mice were weighed every 4 days. The volume of ethanol and water consumed was carefully measured and renewed daily. The ethanol consumed was calculated individually for each mouse, and the values were expressed as g/kg/day. The ratio of ethanol preference was also determined [ethanol preference: ethanol intake/(ethanol intake + water intake)]. Once the 10% ethanol consumption and preference were stabilized for 15 days (or the equivalent period of water access in control animals), vaginal smears were collected in the afternoon for cytological evaluation to identify the mouse estrous cycle stage, selecting only females with high ethanol consumption and preference. Those female mice at the pro-estrus or estrus stage of the cycle were placed into the cage of a singly housed male overnight, and the presence of a vaginal plug was monitored early in the morning. Pregnancy was determined by monitoring weight gain every 2 days and vaginal cytological evaluation. From GD7, throughout all behavioral tests and up to a few hours before sacrifice, mothers previously exposed to voluntary ethanol consumption were administered 3 g/kg of ethanol (p.o., twice a day, every 12 h). The dosage was calculated based on the updated weight of each female mouse, considering variations in weight during the pregnancy, lactation, and post-weaning periods. In contrast, those not exposed to ethanol received tap water (p.o., twice daily) (Figure 1).

Once the offspring had been weaned, ethanol was administered to the dams at a dose of 3 g/kg twice daily (every 12 h), and they were given access to two bottles of ethanol for voluntary consumption until the end of the behavioral tests.

Finally, there were 16 mothers in the ethanol group and 12 dams in the control group after the selection according to ethanol preference (>75%) and the successful completion of the gestational period to term. However, a selection of those dams whose litters were weaned within a narrow time range was performed to avoid temporal differences that could bias the behavioral assessment results (sample sizes are indicated in the figure legends).

### 2.3. BEC

On the weaning day, female mice exposed to voluntary ethanol consumption and ethanol gavage during gestation and lactation received an ethanol dose in the morning (3 g/kg, p.o.). One hour after ethanol administration, trunk blood was collected after rapid decapitation (*n* = 8). According to the manufacturer’s instructions, ethanol concentrations were measured in plasma using an ethanol assay kit (Abcam, Cambridge, MA, USA).

### 2.4. Maternal Care Evaluation

All maternal care evaluation was measured 2 h after the last alcohol intake in the morning. Tests included the undisturbed maternal care test and the retrieval test. The undisturbed maternal care test assessed nest care behavior at PND5. Briefly, nest characteristics were evaluated, and a nest score was assigned according to the criteria proposed by Hess et al., 2008 [23]. For the retrieval test, the pups at PND7 were moved to another cage. After 5 min, two pups, randomly selected without distinction of sex, were placed in the corner of the home cage furthest from the nest, and the latency to retrieval of each pup by the dams was recorded for a maximum of 20 min. Retrieval behavior was defined as the transfer of pups to the nest. If females did not retrieve any pups, the latency to retrieve each pup by females was scored throughout the experimental period (20 min) [24].

### 2.5. Anxiety and Depressive-Like Behavior Evaluation

Behavioral testing began after a week of rest following weaning. Each test was performed with 3 days of rest between each test. The tests were performed two hours after the last ethanol administration. All behavioral tests were conducted in a designated testing room, and animals from both groups were acclimatized for 24 h before testing.

#### 2.5.1. NSFT

This test measures anxiety-induced hyponeophagia, which inhibits food intake in an anxiety-provoking environment [25]. After 24 h of food deprivation, mice were placed in a transparent square cage (40 × 40 × 50 cm) with a single pellet in its center. The latency time before eating was recorded up to a threshold of 5 min. Once the mouse began eating, the food pellet consumption was measured for another 5 min.

#### 2.5.2. TST

It is a widely accepted test for rodent depressive-like behaviors [26]. Mice were suspended by the tail individually at the edge of a lever above the tabletop (at 35 cm) using adhesive tape. The immobility time was measured for 6 min.

### 2.6. Evaluation of Short- and Long-Term Memory

#### SDIA

The apparatus is a 31 × 19 × 15 cm^3^ acrylic box with a platform located next to a grid. Mice were placed on the platform, and their latency to step down on the grid with all four paws was measured; a modified protocol was followed [27]. Immediately after stepping down on the grid during the training session, the animals received a 2.0-s, 0.4-mA scrambled foot shock. Retention tests were procedurally identical, except that no foot shock was given, and the latency to step down in these conditions was taken as a measure of aversive memory. A ceiling of 180 s was imposed; that is, animals with a test latency of more than 180 s were counted as 180 s. Each animal was tested at 1 h (short-term memory) and 24 h after training (long-term memory).

### 2.7. Gene Expression Analyses by qRT-PCR

Relative gene expression of *Crf* in the paraventricular nucleus (PVN), *Nr3c1* in the HIPP, *Oprm1* in the NAcc, *Th* in the ventral tegmental area (VTA), and *Bdnf* in the HIPP were analyzed by real-time polymerase chain reaction (qRT-PCR). At the end of the experimental procedures and 2 h and 30 min after the last alcohol or water administration, female mice were killed by cervical dislocation. Brains were removed from the skull and frozen at −80 °C. These samples were cut in a cryostat (−10 °C), obtaining coronal sections of 500 µm following the atlas of Paxinos and Franklin [28]. They were microdissected following the procedure described by Palkovits and previously performed by our group [29,30]. Total ribonucleic acid (RNA) was extracted with TRI Reagent extraction, and reverse transcription was carried out with the High-Capacity cDNA Reverse Transcription Kit (Thermo Fisher Scientific, Madrid, Spain). Quantitative analyses of the relative gene expression of *Crf* (Mm01293920_s1), *Nr3c1* (Mm00433832_m1), *Oprm1* (Mm01188089_m1), *Th* (Mm00447546_m1), and *Bdnf* (Mm00432069_m1) genes were performed on the StepOne Sequence Detector System (Applied Biosystems, Madrid, Spain) using Taqman assays (Thermo Fisher Scientific, Madrid, Spain). Data for each target gene were normalized to the endogenous reference gene 18S (Mm03928990_g1), and the fold change in target gene expression was determined using the 2^−ΔΔCt^ method [31].

### 2.8. Confocal Immunohistochemistry

Animals were fixed by intracardiac perfusion with 4% (*w*/*v*) paraformaldehyde in 0.1M phosphate buffer (PB), pH 7.4, and post-fixed for 4 h. Coronal sections of the brains were cut with a cryostat (40 µm thick) and stored in 50% (*v*/*v*) glycerol solution at −20 °C until use.

Immunohistochemistry was performed with primary antibodies: mouse anti-S100β (1:1000, Sigma Aldrich, St Louis, MO, USA); rabbit anti-GFAP (1:3000, DAKO, Santa Clara, CA, USA); and rabbit Iba1 (1:3000, DAKO, Santa Clara, CA, USA). For immunofluorescent labeling, after incubating with primary antibodies, brain sections were washed in PBS and incubated for 4h with fluorescent secondary antibodies anti-mouse IgG Alexa 568 and anti-rabbit Alexa 488 (1:1000, Invitrogen, Carlsbad, CA, USA). Sections were later counterstained with Hoechst 33342 (2 µg/mL, Sigma Aldrich, St Louis, MO, USA) to label nuclei and cover-slipped with 70% (*v*/*v*) glycerol mounting medium.

#### Image Analysis

For immunohistochemistry, three separate experiments were run for each immunostaining study. Individual experiments comprised 2 to 4 coronal sections of each animal from each group. Three to five fields were measured from the cerebral area in each coronal section of each animal.

Images were acquired on an inverted microscope with a spinning disk Olympus IX83. Counting and morphometry were performed using Image J for Window (ImageJ bundled with 64-bit Java 8. NIH, http://rsb.info.nih.gov/ij/) software. Brain areas analyzed were the cingulate cortex, the insular cortex, the Amy, and the CA_1_ and CA_3_ regions of the HIPP [28].

Cell counting of S100β+, S100β+/GFAP+ cells, and total Iba-1+ cells was performed. The microscopic areas were counted relative to the area of mm^2^.

To evaluate the percentage of GFAP+ area, the total area of the immunolabeled astrocytes was related to the total area of the corresponding microscopic field (20× primary magnification), rendering a relative area parameter.

Also, Iba-1+ microglial cells were counted according to their morphology: resting, intermediate, activated, and ameboid shape [32,33].

### 2.9. Statistical Analyses

Statistical analyses were performed using the SigmaPlot 11.0 version for all the data. The Student’s *t*-test was used to compare two groups, one-way analysis of variance (ANOVA) to compare 3 or more groups, followed by the Student-Newman-Keuls’ test for the post hoc analyses. Differences were considered significant if the type 1 error or alpha probability was less than 5%.

## 3. Results

### 3.1. Blood Ethanol Concentrations (BEC)

The mean level of ethanol in plasma measured 1 h after the administration (3 g/kg, p.o.) given to the females (mothers) on the day of weaning was 179.02 mg/dl (SEM = 20.16) [20].

### 3.2. Behavioral Evaluations

**Figure 2:**
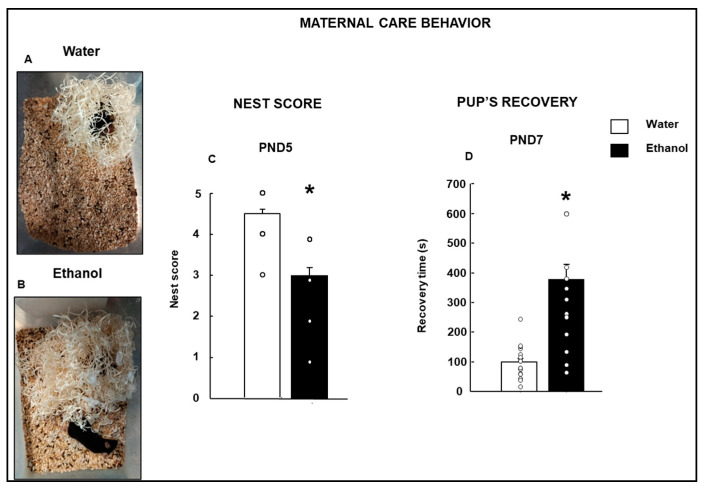
Maternal care evaluation in female mice mothers perinatally exposed to ethanol. Representative pictures of the nests of the Water (**A**) and Ethanol (**B**) groups are shown. At postnatal day 5 (PND5), the nested score was evaluated according to the criteria proposed by Hess et al., 2008 [23] (**C**) (Water: *n* = 12; Ethanol: *n* = 16). Please note that some dots may represent several overlapping values. At PND7, the latency to retrieval pups was measured (**D**) (Water: *n* = 12; Ethanol: *n* = 16). Data were analyzed using a two-tailed Student’s *t*-test. * *p* < 0.01, Ethanol- vs. Water-treated group.

In the comparative results of the undisturbed maternal care test, the nested score was significantly different (Figure 2A–C Student’s *t*-test, t = 3.213; *p* = 0.003 d.f: 28). The nest score of the Ethanol female mice was lower compared to mothers receiving water.

The retrieval test measured the time that mothers spent retrieving two pups. The time of retrieving both pups was significantly higher in Ethanol than in control mothers (Figure 2D, Student’s *t*-test, t = −5.681; *p* = < 0.001; d.f: 32).

#### 3.2.1. Anxiety and Depressive-like Behavior Evaluation

##### Novelty-Suppressed Feeding Test (NSFT)

**Figure 3:**
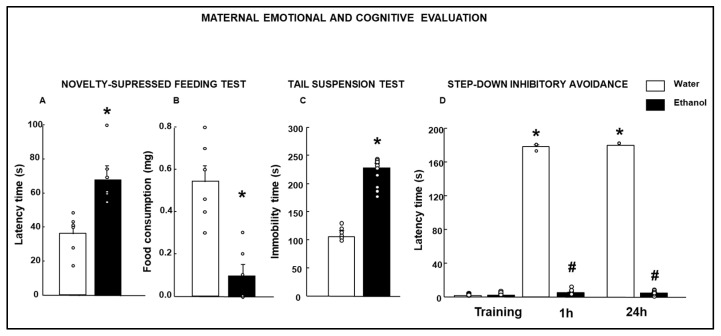
Evaluation of the behavior alterations in female mice perinatally exposed to ethanol or water. The emotional state and aversive stimulus learning performance were evaluated one week after weaning. Anxiety-like behaviors were analyzed by the novelty-suppressed feeding test (Water: *n* = 6; Ethanol: *n* = 8) (latency time: (**A**)). Depressive-like behaviors were evaluated by the novelty-suppressed feeding test (food consumption: (**B**)) and tail suspension test (immobility time: (**C**)) (Water: *n* = 8; Ethanol: *n* = 10). The cognitive performance was evaluated using step-down inhibitory avoidance (latency time to descend the platform at training, 1 h and 24 h: (**D**); (Water: *n* = 8; Ethanol: *n* = 10). Data were analyzed using a two-tailed Student’s *t*-test, * *p* < 0.01, Ethanol- vs. Water-treated group in (**A**–**C**). One-way ANOVA, * *p* < 0.01 Water vs. Water group, and # *p* < 0.01 Ethanol- vs. Water-treated group in (**D**).

In the NSFT paradigm, the results showed a higher latency time in female mothers exposed to ethanol (Figure 3A, *t*-test, t = −3.893; *p* = 0.003; d.f: 10) and reduced food consumption (Figure 3B, Student’s *t*-test, t = 4.759; *p* < 0.001; d.f: 10) compared with controls.

##### Tail Suspension Test (TST)

Mothers exposed to ethanol showed a higher immobility time in the TST paradigm (Figure 3C, Student’s *t*-test, t = 3.912, *p* < 0.001; d.f: 16) than control samples.

#### 3.2.2. Evaluation of Short- and Long-Term Memory

##### Step-Down Inhibitory Avoidance (SDIA)

The results revealed that females perinatally exposed to ethanol showed a reduced latency time when re-exposed at 1 h and 24 h in comparison with the control group (Figure 3D, one-way ANOVA, F (5.53) = 11851.158; # *p* < 0.001). The Water group remembered the aversive stimulus completely (* *p* < 0.001).

### 3.3. Gene Expression Analyses by qRT-PCR

#### 3.3.1. Stress-Related Targets

**Figure 4:**
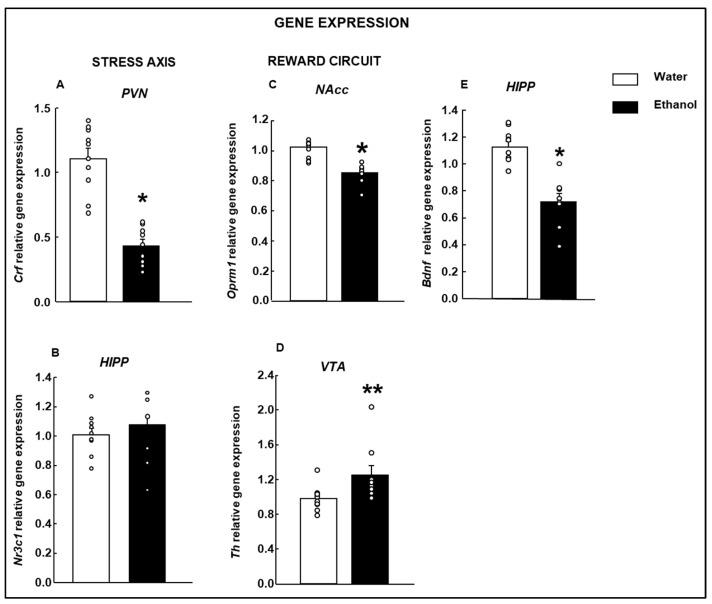
Relative gene expression analyses by qRT-PCR of ethanol-exposed dams during gestation and lactation. Gene expression of corticotropin-releasing factor (Crf; (**A**)) in the paraventricular nucleus (PVN), glucocorticoid receptor (Nr3c1; (**B**)) in the hippocampus (HIPP), mu-opioid receptor 1 (Oprm1; (**C**)) in the nucleus accumbens (NAcc), tyrosine hydroxylase (Th; (**D**)) in the ventral tegmental area (VTA), and brain-derived neurotrophic factor (Bdnf, (**E**)) in the HIPP were evaluated. (Water: *n* = 10; Ethanol: *n* = 8). Data were analyzed using a two-tailed Student’s *t*-test. * *p* < 0.01, ** *p* < 0.05 Ethanol vs. Water group.

Relative gene expression analyses revealed a decrease in gene expression of corticotropin-releasing factor (Crf) in the paraventricular nucleus (PVN) of mother mice (Figure 4A, Student’s *t*-test, t = 7.022, *p* < 0.001; d.f: 17) exposed to ethanol compared with the control group. The glucocorticoid receptor (Nr3c1) was analyzed as an additional target of the stress-axis regulation. Curiously, mice mothers exposed to the ethanol did not show significant differences in Nr3c1 in the HIPP (Figure 4B, Student’s *t*-test, t = −0.687, *p* = 0.502; d.f: 16). However, there was a trend towards increased expression of the receptor in the HIPP of the ethanol-exposed mothers compared to the control group.

#### 3.3.2. Reward Circuitry Targets

*Opioid receptor mu1* (Oprm1) gene expression was significantly reduced in the NAcc of the Ethanol group compared to female mice that received water (Figure 4C: Student’s *t*-test, t: 4.230, *p* < 0.001; d.f.: 17).

The *Tyrosine hydroxylase* (Th) gene expression in the ventral tegmental area (VTA) was increased in the Ethanol females group compared to controls (Figure 3D: Student’s *t*-test, t = −2.340; *p* = 0.032; d.f: 17).

#### 3.3.3. Brain-Derived Neurotrophic Factor (Bdnf)

*Bdnf* gene expression was significantly reduced in the HIPP of females who consumed ethanol during gestation and lactation compared to those who only drank water (Figure 4E: Student’s *t*-test, t = 5.432; *p* < 0.001; d.f: 16).

### 3.4. Immunohistochemistry Analysis

#### 3.4.1. Quantification of Astrocytes

##### S100 Calcium-Binding Protein β-Positive Cells (S100β+) in the Insular Cortex and Amy

**Figure 5:**
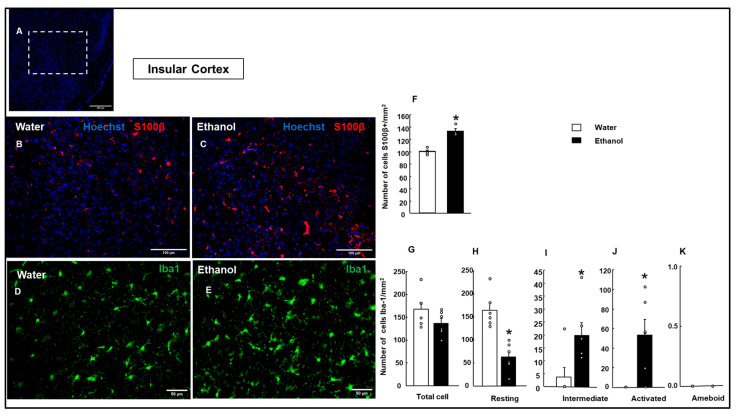
Effect of ethanol exposure on astrocytes and microglial activation in the insular cortex, and Hoechst (blue) staining for visualization of nuclei; the white box indicates the analyzed region (**A**). Representative image of S100β (red) immunostaining at higher magnification of S100β+ cells (red) in Water- and Ethanol-treated groups, with Hoechst (blue) marking staining nuclei (**B**,**C**). Representative images show Iba-1 (green) immunofluorescence, with Hoechst (blue) staining nuclei in both experimental groups in the insular cortex (**D**,**E**). The bar graph quantifies the number of S100β+ cells per mm^2^ in the Water- and Ethanol-treated groups (**F**). Quantification of the total number of microglial cells and their morphological states (resting, intermediate, activated, ameboid, (**G**–**K**)) per area unit. The bar graphs represent the mean ± SEM (*n* = 4–5 per group). Data were analyzed using a two-tailed Student’s *t*-test. * *p* < 0.01 Ethanol vs. Water group.

**Figure 6:**
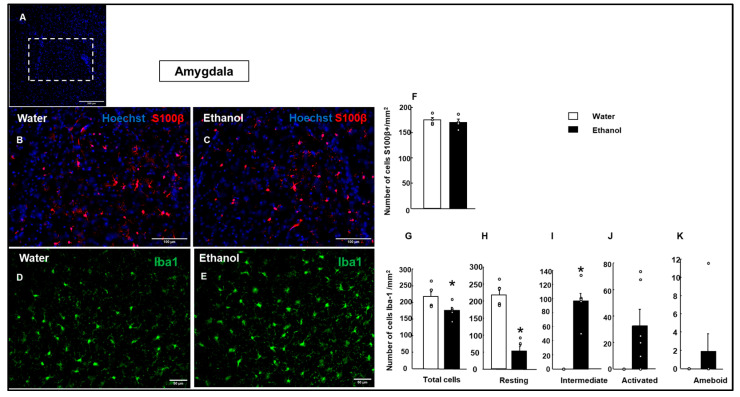
Effects of ethanol exposure on astrocytes and microglial activation in the amygdala (Amy). Representative images of S100β (red) immunofluorescence in the amygdala. A representative amygdala section indicates the area analyzed (white box, (**A**)). Cell nuclei were counterstained with Hoechst (blue) (**B**,**C**). Representative images show Iba1 (green) immunofluorescence in the amygdala (**D**,**E**). Quantification of the number of S100β+ cells per mm^2^ (**F**). Quantification of the total number of microglial cells and their morphological states (resting, intermediate, activated, ameboid (**G**–**K**)) per area unit. The bar graphs represent the mean ± SEM (*n* = 4–5 per group). Data were analyzed using a two-tailed Student’s *t*-test. * *p* < 0.01 Ethanol vs. Water group.

S100β+ cells were measured in the insular cortex and the Amy (Figure 5 and Figure 6). The results showed a significantly higher number of S100β+ cells in the insular cortex in the Ethanol group compared to the Water group (Student’s *t*-test, t = −6.801; *p* < 0.001; d.f: 6) (Figure 5F). Meanwhile, in the Amy (Figure 6F), no significant difference was observed between the Water and Ethanol groups (Student’s *t*-test, *t* = 0.573; *p* = 0.587; d.f: 6), indicating no significant effect of ethanol treatment on S100β+ cell count.

##### S100β+/Anti-Glial Fibrillary Acidic Protein-Positive (GFAP+) Cells in the Cingulate Cortex and HIPP

**Figure 7:**
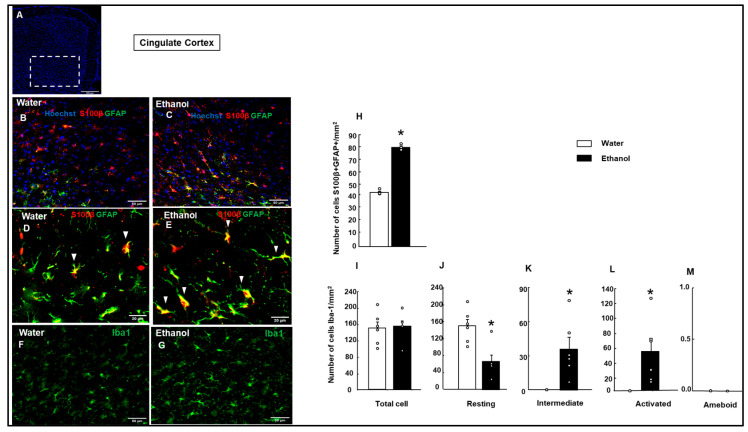
Effects of ethanol exposure on astrocytes and microglial activation in the cingulate cortex: Representative cingulate cortex section indicating the area analyzed (white box, (**A**)). Representative images show GFAP (green) and S100β (red) immunofluorescence in the cingulate cortex. Cell nuclei were counterstained with Hoechst (blue) (**B**,**C**). High-magnification views of S100β/GFAP colocalization are shown for the Water group (**D**) and Ethanol group (**E**). Representative images show Iba-1 (green) immunofluorescence in the insular cortex (**F**,**G**). Quantification of the number of S100β+/GFAP+ cells per mm^2^ (**H**). Quantification of the total number of microglial cells and their morphological states (resting, intermediate, activated, ameboid, (**I**–**M**)) per area unit. The bar graphs represent the mean ± SEM (*n* = 4–5 per group). Data were analyzed using a two-tailed Student’s *t*-test. * *p* < 0.01 Ethanol vs. Water group.

In the cingulate cortex, the analysis revealed a significantly higher number of cells in the Ethanol group compared to the Water group (Student’s *t*-test, t = −25.068; *p* < 0.001; d.f.: 6), on S100β+/GFAP+ cell count (Figure 7B–E,H).

**Figure 8:**
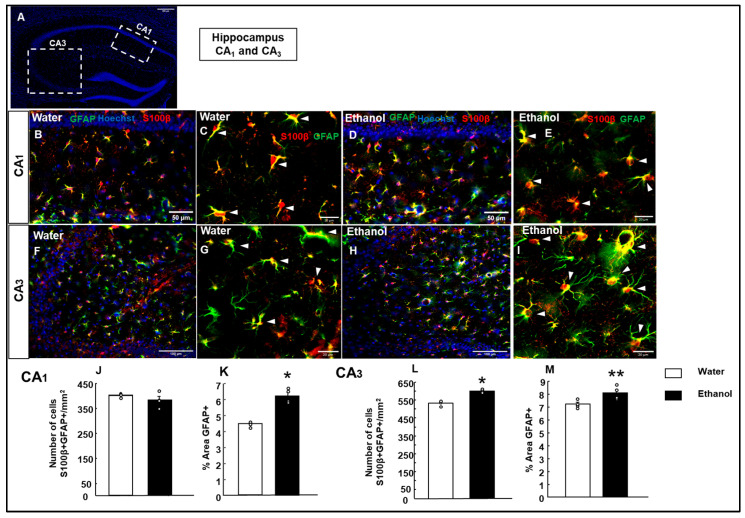
Effects of ethanol exposure on astrocytes in the hippocampal CA_1_ and CA_3_ regions. A representative hippocampal section indicates the areas analyzed (CA_1_ and CA_3_) (**A**). Representative images show GFAP (green) and S100β (red) immunofluorescence in the CA_1_ (**B**–**E**) and CA_3_ (**F**–**I**) regions of the hippocampus from Water- and ETOH-treated groups. Cell nuclei were counterstained with Hoechst (blue). High-magnification views of S100β/GFAP colocalization are shown for the Water group (**C**,**G**) and the Ethanol group (**E**,**I**) in CA_1_ and CA_3_. Quantification of the number of S100β+/GFAP+ cells per mm^2^ and the percentage of GFAP^+^ area is shown for each region (CA_1_ and CA_3_) (**J**–**M**). The bar graphs represent the mean ± SEM (*n* = 4–5 per group). Data were analyzed using a two-tailed Student’s *t*-test. * *p* < 0.01, ** *p* < 0.05 Ethanol vs. Water group.

In the hippocampal CA_1_ region, no significant differences were observed in the number of cells between the experimental and Water groups (Student’s *t*-test, t = −1.343; *p* = 0.228; d.f: 6) (Figure 8J).

In contrast, a significant difference was found in the CA_3_ region (Figure 8L), where the Ethanol group exhibited a higher number of cells compared to the Water group (Student’s *t*-test, t = −7.609; *p* < 0.001; d.f: 6), suggesting that ethanol exposure significantly increased the number of S100β+/GFAP+ cells in the CA_3_ region of the HIPP.

##### GFAP+ Cells’ Relative Area in the HIPP

Astrogliosis was also analyzed by measuring the relative area covered by GFAP+ astrocytes. The analysis revealed a significant increase in the relative area of GFAP+ astrocytes in the Ethanol group compared to the Water group (Student’s *t*-test, t = −7.542; *p* < 0.001; d.f: 6) in the CA_1_ hippocampal area (Figure 8K).

Similarly, a significant difference was observed in the CA_3_ region (Figure 8M), where the Ethanol group showed a larger relative astrocytic area compared to the Water group (Student’s *t*-test, t = −2.934; *p* = 0.026; d.f: 6), suggesting that ethanol exposure also significantly increased the relative area of GFAP+ astrocytes.

#### 3.4.2. Microglial Cell Reaction

**Figure 9:**
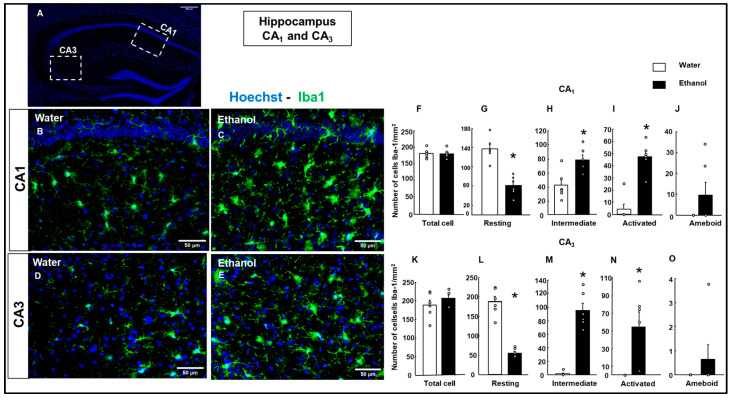
Effect of ethanol exposure on microglial activation in the hippocampal regions CA_1_ and CA_3_. A representative hippocampal section indicates the areas analyzed (CA_1_ and CA_3_, (**A**)). Representative images show Iba-1 (green) immunofluorescence in the CA_1_ (**B**,**C**) and CA_3_ (**D**,**E**) regions of the hippocampus from Water- and ETOH-treated groups. Cell nuclei were counterstained with Hoechst (blue). Quantification of the total number of microglial cells and their morphological states (resting, intermediate, activated, and ameboid) is shown for each region (CA_1_ and CA_3_) (**F**–**O**) per area unit. The bar graphs represent the mean ± SEM (*n* = 4–5 per group). Data were analyzed using a two-tailed Student’s *t*-test. * *p* < 0.01 Ethanol vs. Water group.

Immunostaining with anti-ionized calcium-binding adapter molecule 1 (Iba-1) was used to study microglia. Total Iba-1+ microglial cells were measured in the insular cortex (Figure 5), Amy (Figure 6), cingulate cortex (Figure 7), and HIPP (Figure 9). In addition, the morphological microglial phenotype was analyzed, measuring resting, intermediate, activated, and ameboid shape Iba-1+ cells.

In the insula, total microglial Iba-1+ cells did not show a significant difference between the Water and Ethanol groups (Figure 5G; Student’s *t*-test, t = 1.618; *p* = 0.137; d.f: 10). Resting cells showed a significant decrease in the Ethanol group (Figure 5H; Student’s *t*-test, t = 1.618; *p* = 5.036; *p* < 0.001; d.f: 10). Intermediate and activated microglial cells were significantly increased in the Ethanol group compared to the Water group (Figure 5I; intermediate microglia: Student’s *t*-test, t = −2.639; *p* = 0.025; d.f: 10; Figure 5J; activated microglia: Student’s *t*-test, t = −3.376; *p* = 0.007; d.f: 10). In contrast, no ameboid microglial cells were found in either the control or Ethanol groups (Figure 5K).

In the Amy, the analysis of microglial cells revealed significant differences between the Water and Ethanol groups in total Iba-1+ microglial cells (Figure 6G; Student’s *t*-test, t = 2.539; *p* = 0.029; d.f: 10). Resting Iba-1+ microglial cells were significantly reduced in the Ethanol group compared to the Water group (Figure 6H; Student’s *t*-test, t = 8. 736; *p* < 0.001; d.f: 10). In contrast, intermediate Iba-1+ microglial cells showed a significant increase in the Ethanol group (Figure 6I; Student’s *t*-test, t = −8.929, *p* < 0.001; d.f: 10). No significant differences were observed in activated Iba-1+ microglial cells between the Water and Ethanol groups (Figure 6J; Student’s *t*-test, t = −1. 738; *p* = 0.113; d.f: 10). Similarly, ameboid Iba-1+ microglial cells showed no significant difference between the groups (Figure 6K; Student’s *t*-test, t = 1.000; *p* = 0.341; d.f: 10).

In the cingulate cortex, total microglial Iba-1+ cells did not show a significant difference between the Water and Ethanol groups (Figure 7I; Student’s *t*-test, t = −0.220; *p* = 0.830; d.f: 10). In this cortical area, the resting cells showed a significant decrease in the Ethanol group (Figure 7J, Student’s *t*-test, t = −3.472; *p* = 0.004; d.f: 10). In contrast, intermediate and activated microglial cells showed a significant increase in the Ethanol group (Figure 7K; intermediate microglia: Student’s *t*-test, t = 3.769; *p* = 0.006; d.f: 10; Figure 7L; activated microglia: Student’s *t*-test, t = −2.989; *p* = 0.014; d.f: 10; for intermediate and activated microglial cells, respectively). Moreover, ameboid microglial cells did not differ significantly between the Water and Ethanol groups (Figure 7M, Student’s *t*-test, t = 0.000; *p* = 1.000; d.f: 10). The total number of Iba-1+ microglial cells in the CA_1_ region did not show a significant difference between the Water and Ethanol groups (Figure 9F, Student’s *t*-test, t = 0.000; *p* = 0.194; d.f: 10). Resting Iba-1+ microglial cells were significantly reduced in the Ethanol group compared to the Water group (Figure 9G; Student’s *t*-test, t = 5.872; *p* < 0.001; d.f: 10). In contrast, both intermediate and activated microglial cells showed a significant increase in the Ethanol group (Figure 9H; intermediate microglia: Student’s *t*-test, t = −3.398; *p* = 0.007; d.f: 10; Figure 9I; activated microglia: Student’s *t*-test, t = −6.724; *p* < 0.001; d.f.: 10). Ameboid Iba-1+ microglial cells showed no significant difference between the groups (Figure 9J; Student’s *t*-test, t = −1.544; *p* = 0.154; d.f: 10).

Similarly, the total number of Iba-1+ microglial cells in the CA_3_ region did not differ significantly between groups (Figure 9K, Student’s *t*-test, t = −0.982; *p* = 0.349; d.f: 10). Resting microglial cells were significantly decreased in the Ethanol group (Figure 9L, Student’s *t*-test, t = 8.869; *p* < 0.001; d.f: 10). Conversely, intermediate and activated microglial cells were significantly increased in the Ethanol group compared to the Water group (Figure 9M; intermediate microglia: Student’s *t*-test, t = −8.699; *p* < 0.001; d.f: 10; Figure 9N; activated microglia: Student’s *t*-test, t = −3.194; *p* = 0.010; d.f: 10). No significant differences were observed in ameboid microglial cells between the groups (Figure 9O, Student’s *t*-test, t = −1.000; *p* = 0.341, d.f: 10).

## 4. Discussion

The results of this study indicate that alcohol consumption during gestation and lactation is associated with measurable alterations in maternal behavior and neurobiological function, which may adversely affect offspring care and behavioral outcomes. The results included in this study indicate that (1) Mice mothers exposed to ethanol showed a significant reduction in the nested score, (2) The time of retrieving both pups was significantly higher in ethanol mothers compared to control mothers, (3) Female mice exposed to ethanol showed significant anxiety- and depressive-like behaviors as well as short and long term memories disturbances, (4) These behavioral changes were accompanied with gene expression alterations in different brain targets related with neuroplasticity, stress regulation and reward circuitry (*Bdnf*, *Crf*, *Nr3c1*, *Oprm1*, and *Th*), and (5) Microglial and astroglial changes were detected in areas closely related with cognitive, anxiety and depressive processes such as the HIPP, insula, Amy, and cingulate cortex.

Studying the effects of alcohol exposure in female mice consuming ethanol is crucial because it allows for the identification of changes in behavior, learning, and aversive memory processing that may be associated with alterations in maternal care, potentially influencing offspring development. Additionally, this is the only experimental model with these specific characteristics that has evaluated maternal care, as well as behavioral and neuroplasticity-related changes, in female mice exposed to ethanol during both gestation and lactation. Using a recently validated FASD model, we assessed maternal behavior and neuroplasticity-related changes in mouse dams [20]. Several animal models have been used to simulate the development of FASD. These models involve exposing pregnant rodents or their offspring to different alcohol concentrations by oral gavage or subcutaneous administration at various stages of gestation or postnatal development [34,35,36,37,38,39,40,41,42,43]. Our model varied considerably, as it included specific features not found in previously published models, which may differentially modify the results [20]. This is the first study considering the previous alcohol consumption for developing the animal model of FASD, avoiding any period of abstinence in females between both gavage administrations. However, the voluntary ethanol consumption using these bottles was completely null, indicating the absence of abstinence periods. One hour after the last ethanol administration, ethanol concentrations in plasma were elevated (179.02 ± 20.16 mg/dL), causing some problems with motor coordination in the females [34,41]. These were especially noticeable at the beginning of ethanol gavage administration and diminished throughout treatment [20].

The present study observed alterations in maternal care behaviors toward the pups. At PND4, new nesting material was placed on the cage, and at PND5, nest construction was scored based on previous work [23]. Ethanol-exposed female mice built nests that received lower scores than Water-exposed mice during pregnancy and lactation until sacrifice. The same occurred with the pup’s retrieval. Some ethanol-exposed female mice did not recover their offspring within the maximum time allowed for the test. In concordance with these results, Workman et al. reported a significant disruption in maternal care with reduced nursing posture using ad libitum 36% ethanol intake [44]. However, some works found no difference in the time taken for offspring recovery at different postnatal ages in a mouse model of prenatal ethanol exposure using the voluntary consumption paradigm compared with the control group [45,46,47].

In our study, drawing upon previous epidemiological findings, we developed a model that reflects the substantial alcohol consumption observed among women before and during pregnancy. Consequently, we aimed to design a model with a high degree of translational relevance. This model differs significantly from previously established paradigms, incorporating several critical features: (1) a history of pregestational alcohol consumption, (2) exposure to alcohol during both gestation and lactation, (3) administration of high doses of ethanol, and (4) significant alterations in both behavior and biomarkers [48]. These parameters resulted in elevated blood ethanol concentrations, which may account for the distinct maternal caregiving behaviors observed in ethanol-exposed dams relative to controls [3,4,48]. The elevated blood levels observed in the ethanol-exposed dams are a limitation on the maternal caregiving behaviors. However, we contend that these elevated ethanol levels provide compelling evidence of the detrimental effects of high blood alcohol concentrations on maternal behavior. Given the current prevalence of child maltreatment and the morbidity associated with maternal alcohol abuse, we propose that this model more accurately represents contemporary epidemiological patterns. Furthermore, despite the high ethanol dosage administered two hours before behavioral testing, ethanol-exposed females demonstrated preserved motor function. For example, latency to descend from the platform in the Step-Down Inhibitory Avoidance (SDIA) task was comparable between ethanol- and water-exposed groups (see Figure 2D). These findings suggest that the observed behavioral differences are not attributable to acute ethanol intoxication, but rather to the effects of chronic ethanol exposure.

Additionally, some authors have suggested that the deficiency in maternal care is due to changes in the pups’ behavior toward their mothers. Rat pups prenatally exposed to alcohol have reduced ultrasonic vocalizations, which could limit alcohol-exposed offspring’s ability to elicit the same levels of maternal care as controls [49,50].

Nevertheless, it was demonstrated in some clinical studies that mothers exposed perinatally to ethanol presented altered maternal behavior [8,51,52]. Our results showed anxiety- and depressive-like behaviors in mothers exposed to ethanol when exposed to the NSFT and TST paradigms. Interestingly, our gene expression findings suggested that Ethanol-treated mothers presented an alteration in the HPA axis regulation. Indeed, the relative gene expression of *Crf* in the PVN and *Nr3c1* in the HIPP was evaluated to detect changes that may help to elucidate, at least in part, the anxiety and depressive-like behavior caused by PAE. Although there was a tendency to increase, a difference in the glucocorticoid receptor was not detected. However, a significant reduction in *Crf* gene expression in Ethanol mothers was found. It is important to note that the Ethanol and Water-exposed groups underwent identical procedures performed simultaneously. Therefore, any potential stress-related effects induced by the behavioral tests would have affected both groups equally, with the control group as the reference for baseline comparisons. Previous studies reported that rats chronically exposed to alcohol during the postpartum period display lower corticosterone concentrations than controls and no changes in adrenal mass [53]. Moreover, it has been found that stress-induced HPA activity is usually suppressed during the postpartum period [54,55]. In our case, the brains were obtained approximately four weeks after delivery, which may suggest that in the long term, only changes in some specific axis targets are observed. The hormones involved during the postpartum period may protect against the significant changes that alcohol can produce in the HPA axis. However, future research should be conducted to confirm this hypothesis.

Cognitive impairment has been reported after chronic consumption of ethanol. Previous work showed that gene expression of *Bdnf* in the HIPP and plasma levels of *Bdnf* were decreased in ethanol-exposed rats, and a correlation between the *Bdnf* level and cognitive impairment in the novel object recognition test has been found [56]. Indeed, the BDNF-TrkB pathway is central to activity-dependent synaptic plasticity associated with learning and memory [57]. In our case, mothers showed impaired aversive memory in both the short and long term. In addition, gene expression analyses revealed that Ethanol mothers presented decreased *Bdnf* gene expression in the HIPP as described above.

Alcohol exposure has been associated with significant alterations in glial cell function, particularly affecting astrocytes and microglia. Astrocytes, identified by GFAP and S100β, and microglia, labeled by Iba-1, play an essential role in regulating central nervous system homeostasis [58,59].

Microglia cells are activated in response to ethanol, as indicated by increased Iba-1 expression, showing alcohol-induced neuroinflammation [60,61]. An inflammatory process in the HIPP was detected in mothers who consume alcohol during gestation and lactation. The number of activated microglial cells significantly increased in the CA_1_ and CA_3_ regions of the HIPP. Moreover, there was an increase in the percentage of area occupied by astroglial cells in CA_1_ and CA_3_, and the number of GFAP+/S100β+ cells increased only in CA_3_. The upregulation of GFAP often correlates with elevated S100β levels, suggesting concurrent astrocytic responses to ethanol exposure [62,63]. S100β protein also has a role in modulating synaptic plasticity and hippocampal-dependent memory. Overexpression of S100β in mice is associated with impaired spatial memory in the Morris water maze [64]. In addition, several studies also show that chronic alcohol consumption produces the pro-inflammatory phenotype of microglia and astroglia cells [65,66,67,68]. Microglial or astroglial changes in the HIPP suggest that this inflammatory process could be associated with memory impairment [68,69]. Our results support the idea that memory impairment was linked to changes in astroglia and microglia cells in the HIPP. This is evidenced by comparing mothers who consumed only water during pregnancy and lactation, showing no signs of an inflammatory process in the HIPP.

The reward circuit is also affected by ethanol consumption in mouse mothers. Specifically, real-time PCR gene expression studies showed lower *Oprm1* gene expression in the NAcc of mouse mothers who consumed ethanol during pregnancy and lactation compared to the control group. However, *Th* gene expression increased in female Ethanol mothers in the VTA group compared to the Water group. Therefore, alcohol exposure during pregnancy and lactation may result in functional changes in the mesolimbic dopaminergic system. Notably, after chronic ethanol administration, dopaminergic pathways originating in the VTA and projecting to the NAcc undergo functional changes [70,71,72,73]. In addition, in the insula, which receives inputs from the NAcc, it was found that most microglial cells are activated, and there is an increase in the number of S100β-immune responsive cells in mothers who consume ethanol compared with the control group. S100β, at high concentrations, can act as a pro-inflammatory and neurotoxic molecule, promoting cellular damage rather than neuroprotection. Elevated S100β protein may exacerbate neuroinflammation and neuronal dysfunction in this area [74]. In the cingulate cortex, an area with essential functions in reward circuitry, there was a significant increase in activated microglial cells and S100β/GFAP positive cells in the Ethanol group compared with the control group. A significant increase in GFAP and S100β may indicate reactive gliosis, an astrocytic response to brain injury, inflammation, or neurodegeneration [75]. It is important to note that craving and relapses in alcohol-dependent patients are linked to changes in the cingulate cortex [76,77]. However, in Amy, a brain region implicated in anxiety, stress-related disorders, and the reinforcing effects of alcohol, there were no differences in the numbers of S100β-inmunoresponsive cells between the Ethanol and Water groups. In addition, the most significant number of microglial cells was in the intermediate state in the Ethanol female mice. Thus, histological analysis revealed no detectable inflammatory response in this area.

The amygdala is a brain structure influenced by sex hormone signaling [78,79], and several studies have reported sex-specific effects of stress and alcohol consumption on microglial number and function within this region. [80,81,82]. Estrogens produce anti-inflammatory effects by regulating microglia, possibly contributing to their neuroprotective effects [83,84]. Estrogens may, therefore, contribute to the absence of inflammation in this area. Future studies are needed to assess the neurochemical mechanisms involved.

## 5. Conclusions

In conclusion, this translational animal model highlights the complications that alcohol causes in mothers who consume alcohol during pregnancy, breastfeeding, and post-weaning periods. These problems range from deficits in offspring care to anxiety, depressive-like behavior, and memories of aversive stimulus disturbances that are accompanied by significant brain alterations in nuclei related to the stress axis and the limbic system, also leading to inflammatory processes of the brain reflected in the evident microgliosis and astroglia alteration in distinct brain regions. Therefore, this study sheds light on the negative behavioral and neurobiological consequences of maternal alcohol consumption, suggesting that more preventive and adequate measures are necessary to discourage mothers from using alcohol during this critical life period that affects themselves and their offspring.

## Data Availability

The original contributions presented in this study are included in the article. Further inquiries can be directed to the corresponding author.

## Abbreviations

Amy	Amygdala
AUD	Alcohol use disorders
Bdnf	Brain-derived neurotrophic factor
BEC	Blood ethanol concentrations
Crf	Corticotropin-releasing factor
FASD	Fetal alcohol spectrum disorder
GD	Gestational day
GFAP	Anti-glial fibrillary acidic protein
HIPP	Hippocampus
Iba-1	Anti-ionized calcium-binding adapter molecule 1
NAcc	Nucleus accumbens
Nr3c1	Glucocorticoid receptor
NSFT	Novelty-suppressed feeding test
Oprm1	Opioid receptor mu1
PAE	Perinatal alcohol exposure
PND	Postnatal day
PVN	Paraventricular nucleus
S100β	S100 calcium-binding protein β
SDIA	Step-down inhibitory avoidance
Th	Tyrosine hydroxylase
TST	Tail suspension test
VTA	Ventral tegmental area

## References

[1] Broccia M., Munch A., Hansen B.M., Sorensen K.K., Larsen T., Strandberg-Larsen K., Gerds T.A., Torp-Pedersen C., Kesmodel U.S. (2023). Heavy prenatal alcohol exposure and overall morbidities: A Danish nationwide cohort study from 1996 to 2018. Lancet Public Health.

[2] May P.A., de Vries M.M., Marais A.S., Kalberg W.O., Buckley D., Hasken J.M., Abdul-Rahman O., Robinson L.K., Manning M.A., Seedat S. (2022). The prevalence of fetal alcohol spectrum disorders in rural communities in South Africa: A third regional sample of child characteristics and maternal risk factors. Alcohol Clin. Exp. Res..

[3] May P.A., Chambers C.D., Kalberg W.O., Zellner J., Feldman H., Buckley D., Kopald D., Hasken J.M., Xu R., Honerkamp-Smith G. (2018). Prevalence of Fetal Alcohol Spectrum Disorders in 4 US Communities. JAMA.

[4] Roberts S.C.M., Schulte A., Zaugg C., Leslie D.L., Corr T.E., Liu G. (2023). Association of Pregnancy-Specific Alcohol Policies with Infant Morbidities and Maltreatment. JAMA Netw. Open.

[5] Lange S., Probst C., Gmel G., Rehm J., Burd L., Popova S. (2017). Global Prevalence of Fetal Alcohol Spectrum Disorder Among Children and Youth: A Systematic Review and Meta-analysis. JAMA Pediatr..

[6] Popova S., Dozet D., Akhand Laboni S., Brower K., Temple V. (2022). Why do women consume alcohol during pregnancy or while breastfeeding?. Drug Alcohol Rev..

[7] The Centre for Addiction and Mental Health (CAMH) Alcohol Use: Managing Alcohol Use in Pregnancy. https://www.camh.ca/en/professionals/treating-conditions-and-disorders/alcohol-use/alcohol-use---treatment/treatment---managing-alcohol-use-in-pregnancy.

[8] Nonacs R.M., Queenan J.T., Spong C.Y., Lockwood C.J. (2020). Alcohol Use in Pregnancy and Lactation. Protocols for High-Risk Pegnancies: An Evidence-Based Approach.

[9] White A.M. (2003). What happened? Alcohol, memory blackouts, and the brain. Alcohol Res. Health.

[10] Magrys S.A., Olmstead M.C. (2014). Alcohol intoxication alters cognitive skills mediated by frontal and temporal brain regions. Brain Cogn..

[11] Zorumski C.F., Mennerick S., Izumi Y. (2014). Acute and chronic effects of ethanol on learning-related synaptic plasticity. Alcohol.

[12] Epstein E.E., Fischer-Elber K., Al-Otaiba Z. (2007). Women, aging, and alcohol use disorders. J. Women Aging.

[13] Mumenthaler M.S., Taylor J.L., O’Hara R., Yesavage J.A. (1999). Gender differences in moderate drinking effects. Alcohol Res. Health.

[14] Frezza M., di Padova C., Pozzato G., Terpin M., Baraona E., Lieber C.S. (1990). High blood alcohol levels in women. The role of decreased gastric alcohol dehydrogenase activity and first-pass metabolism. N. Engl. J. Med..

[15] Dannenhoffer C.A., Robertson M.M., Macht V.A., Mooney S.M., Boettiger C.A., Robinson D.L. (2021). Chronic alcohol exposure during critical developmental periods differentially impacts persistence of deficits in cognitive flexibility and related circuitry. Int. Rev. Neurobiol..

[16] Sircar R., Basak A.K., Sircar D. (2009). Repeated ethanol exposure affects the acquisition of spatial memory in adolescent female rats. Behav. Brain Res..

[17] Teixeira F.B., Santana L.N., Bezerra F.R., De Carvalho S., Fontes-Junior E.A., Prediger R.D., Crespo-Lopez M.E., Maia C.S., Lima R.R. (2014). Chronic ethanol exposure during adolescence in rats induces motor impairments and cerebral cortex damage associated with oxidative stress. PLoS ONE.

[18] Charlton A.J., May C., Luikinga S.J., Burrows E.L., Hyun Kim J., Lawrence A.J., Perry C.J. (2019). Chronic voluntary alcohol consumption causes persistent cognitive deficits and cortical cell loss in a rodent model. Sci. Rep..

[19] Garcia-Moreno L.M., Cimadevilla J.M. (2012). Acute and chronic ethanol intake: Effects on spatial and non-spatial memory in rats. Alcohol.

[20] Gasparyan A., Navarro D., Navarrete F., Austrich-Olivares A., Scoma E.R., Hambardikar V.D., Acosta G.B., Solesio M.E., Manzanares J. (2023). Cannabidiol repairs behavioral and brain disturbances in a model of fetal alcohol spectrum disorder. Pharmacol. Res..

[21] Kilkenny C., Browne W.J., Cuthill I.C., Emerson M., Altman D.G. (2010). Improving bioscience research reporting: The ARRIVE guidelines for reporting animal research. J. Pharmacol. Pharmacother..

[22] Percie du Sert N., Hurst V., Ahluwalia A., Alam S., Avey M.T., Baker M., Browne W.J., Clark A., Cuthill I.C., Dirnagl U. (2020). The ARRIVE guidelines 2.0: Updated guidelines for reporting animal research. PLoS Biol..

[23] Hess S.E., Rohr S., Dufour B.D., Gaskill B.N., Pajor E.A., Garner J.P. (2008). Home improvement: C57BL/6J mice given more naturalistic nesting materials build better nests. J. Am. Assoc. Lab. Anim. Sci..

[24] Fujisaki M., Nakamura A., Muroi Y., Ishii T. (2020). Oxytocin in the dorsal raphe nucleus antagonizes the inhibition of maternal care induced by food deprivation. Horm. Behav..

[25] Bodnoff S.R., Suranyi-Cadotte B., Aitken D.H., Quirion R., Meaney M.J. (1988). The effects of chronic antidepressant treatment in an animal model of anxiety. Psychopharmacology.

[26] Vaugeois J.M., Passera G., Zuccaro F., Costentin J. (1997). Individual differences in response to imipramine in the mouse tail suspension test. Psychopharmacology.

[27] Izquierdo I., Izquierdo L.A., Barros D.M., Mello e Souza T., de Souza M.M., Quevedo J., Rodrigues C., Sant’Anna M.K., Madruga M., Medina J.H. (1998). Differential involvement of cortical receptor mechanisms in working, short-term and long-term memory. Behav. Pharmacol..

[28] Paxinos G., Franklin K.B.J. (2004). The Mouse Brain in Stereotaxic Coordinates.

[29] Palkovits M. (1983). Punch sampling biopsy technique. Methods Enzymol..

[30] Navarrete F., Perez-Ortiz J.M., Manzanares J. (2012). Pregabalin- and topiramate-mediated regulation of cognitive and motor impulsivity in DBA/2 mice. Br. J. Pharmacol..

[31] Livak K.J., Schmittgen T.D. (2001). Analysis of relative gene expression data using real-time quantitative PCR and the 2(-Delta Delta C(T)) Method. Methods.

[32] Green T.R.F., Rowe R.K. (2024). Quantifying microglial morphology: An insight into function. Clin. Exp. Immunol..

[33] Vidal-Itriago A., Radford R.A.W., Aramideh J.A., Maurel C., Scherer N.M., Don E.K., Lee A., Chung R.S., Graeber M.B., Morsch M. (2022). Microglia morphophysiological diversity and its implications for the CNS. Front. Immunol..

[34] Garcia-Baos A., Puig-Reyne X., Garcia-Algar O., Valverde O. (2021). Cannabidiol attenuates cognitive deficits and neuroinflammation induced by early alcohol exposure in a mice model. Biomed. Pharmacother..

[35] Brocardo P.S., Boehme F., Patten A., Cox A., Gil-Mohapel J., Christie B.R. (2012). Anxiety- and depression-like behaviors are accompanied by an increase in oxidative stress in a rat model of fetal alcohol spectrum disorders: Protective effects of voluntary physical exercise. Neuropharmacology.

[36] Banuelos C., Gilbert R.J., Montgomery K.S., Fincher A.S., Wang H., Frye G.D., Setlow B., Bizon J.L. (2012). Altered spatial learning and delay discounting in a rat model of human third trimester binge ethanol exposure. Behav. Pharmacol..

[37] Hunt P.S., Barnet R.C. (2015). An animal model of fetal alcohol spectrum disorder: Trace conditioning as a window to inform memory deficits and intervention tactics. Physiol. Behav..

[38] Huebner S.M., Tran T.D., Rufer E.S., Crump P.M., Smith S.M. (2015). Maternal iron deficiency worsens the associative learning deficits and hippocampal and cerebellar losses in a rat model of fetal alcohol spectrum disorders. Alcohol. Clin. Exp. Res..

[39] Holman P.J., Raineki C., Chao A., Grewal R., Haghighat S., Fung C., Morgan E., Ellis L., Yu W., Weinberg J. (2021). Altered social recognition memory and hypothalamic neuropeptide expression in adolescent male and female rats following prenatal alcohol exposure and/or early-life adversity. Psychoneuroendocrinology.

[40] Endres M., Toso L., Roberson R., Park J., Abebe D., Poggi S., Spong C.Y. (2005). Prevention of alcohol-induced developmental delays and learning abnormalities in a model of fetal alcohol syndrome. Am. J. Obs. Obstet. Gynecol..

[41] Cantacorps L., Gonzalez-Pardo H., Arias J.L., Valverde O., Conejo N.M. (2018). Altered brain functional connectivity and behaviour in a mouse model of maternal alcohol binge-drinking. Prog. Neuropsychopharmacol. Biol. Psychiatry.

[42] Ieraci A., Herrera D.G. (2020). Early Postnatal Ethanol Exposure in Mice Induces Sex-Dependent Memory Impairment and Reduction of Hippocampal NMDA-R2B Expression in Adulthood. Neuroscience.

[43] Lawrence R.C., Otero N.K., Kelly S.J. (2012). Selective effects of perinatal ethanol exposure in medial prefrontal cortex and nucleus accumbens. Neurotoxicol. Teratol..

[44] Workman J.L., Raineki C., Weinberg J., Galea L.A.M. (2015). Alcohol and pregnancy: Effects on maternal care, HPA axis function, and hippocampal neurogenesis in adult females. Psychoneuroendocrinology.

[45] Allan A.M., Chynoweth J., Tyler L.A., Caldwell K.K. (2003). A mouse model of prenatal ethanol exposure using a voluntary drinking paradigm. Alcohol Clin. Exp. Res..

[46] Brady M.L., Allan A.M., Caldwell K.K. (2012). A limited access mouse model of prenatal alcohol exposure that produces long-lasting deficits in hippocampal-dependent learning and memory. Alcohol Clin. Exp. Res..

[47] Kleiber M.L., Wright E., Singh S.M. (2011). Maternal voluntary drinking in C57BL/6J mice: Advancing a model for fetal alcohol spectrum disorders. Behav. Brain Res..

[48] Gosdin L.K., Deputy N.P., Kim S.Y., Dang E.P., Denny C.H. (2022). Alcohol Consumption and Binge Drinking During Pregnancy Among Adults Aged 18–49 Years—United States, 2018–2020. MMWR Morb. Mortal. Wkly. Rep..

[49] Chen J.S., Driscoll C.D., Riley E.P. (1982). Ontogeny of suckling behavior in rats prenatally exposed to alcohol. Teratology.

[50] Kehoe P., Shoemaker W. (1991). Opioid-dependent behaviors in infant rats: Effects of prenatal exposure to ethanol. Pharmacol. Biochem. Behav..

[51] O’Connor M.J., Paley B. (2006). The relationship of prenatal alcohol exposure and the postnatal environment to child depressive symptoms. J. Pediatr. Psychol..

[52] Pearson R.M., Heron J., Melotti R., Joinson C., Evans J. (2012). The impact of alcohol use during pregnancy on maternal responses after birth. Arch. Womens Ment. Health.

[53] Mahieu H.F., Schutte H.K. (1989). New surgical techniques for voice improvement. Arch. Otorhinolaryngol..

[54] Allolio B., Hoffmann J., Linton E.A., Winkelmann W., Kusche M., Schulte H.M. (1990). Diurnal salivary cortisol patterns during pregnancy and after delivery: Relationship to plasma corticotrophin-releasing-hormone. Clin. Endocrinol..

[55] Neumann I.D., Johnstone H.A., Hatzinger M., Liebsch G., Shipston M., Russell J.A., Landgraf R., Douglas A.J. (2004). Attenuated neuroendocrine responses to emotional and physical stressors in pregnant rats involve adenohypophysial changes. J. Physiol..

[56] Silva-Pena D., Garcia-Marchena N., Alen F., Araos P., Rivera P., Vargas A., Garcia-Fernandez M.I., Martin-Velasco A.I., Villanua M.A., Castilla-Ortega E. (2019). Alcohol-induced cognitive deficits are associated with decreased circulating levels of the neurotrophin BDNF in humans and rats. Addict. Biol..

[57] Yin J.B., Wu H.H., Dong Y.L., Zhang T., Wang J., Zhang Y., Wei Y.Y., Lu Y.C., Wu S.X., Wang W. (2014). Neurochemical properties of BDNF-containing neurons projecting to rostral ventromedial medulla in the ventrolateral periaqueductal gray. Front. Neural Circuits.

[58] Chen Z., Yuan Z., Yang S., Zhu Y., Xue M., Zhang J., Leng L. (2023). Brain Energy Metabolism: Astrocytes in Neurodegenerative Diseases. CNS Neurosci. Ther..

[59] Wright-Jin E.C., Gutmann D.H. (2019). Microglia as Dynamic Cellular Mediators of Brain Function. Trends Mol. Med..

[60] Portis S.M., Haass-Koffler C.L. (2020). New Microglial Mechanisms Revealed in Alcohol Use Disorder: How Does That Translate?. Biol. Psychiatry.

[61] Li Q., Liu D., Pan F., Ho C.S.H., Ho R.C.M. (2019). Ethanol Exposure Induces Microglia Activation and Neuroinflammation through TLR4 Activation and SENP6 Modulation in the Adolescent Rat Hippocampus. Neural Plast..

[62] Janigro D., Mondello S., Posti J.P., Unden J. (2022). GFAP and S100B: What You Always Wanted to Know and Never Dared to Ask. Front. Neurol..

[63] Vizuete A.F.K., Mussulini B.H., Zenki K.C., Baggio S., Pasqualotto A., Rosemberg D.B., Bogo M.R., de Oliveira D.L., Rico E.P. (2022). Prolonged ethanol exposure alters glutamate uptake leading to astrogliosis and neuroinflammation in adult zebrafish brain. Neurotoxicology.

[64] Gerlai R., Wojtowicz J.M., Marks A., Roder J. (1995). Overexpression of a calcium-binding protein, S100 beta, in astrocytes alters synaptic plasticity and impairs spatial learning in transgenic mice. Learn. Mem..

[65] Melbourne J.K., Thompson K.R., Peng H., Nixon K. (2019). Its complicated: The relationship between alcohol and microglia in the search for novel pharmacotherapeutic targets for alcohol use disorders. Prog. Mol. Biol. Transl. Sci..

[66] Crews F.T., Zou J., Coleman L.G. (2021). Extracellular microvesicles promote microglia-mediated pro-inflammatory responses to ethanol. J. Neurosci. Res..

[67] Alfonso-Loeches S., Urena-Peralta J., Morillo-Bargues M.J., Gomez-Pinedo U., Guerri C. (2016). Ethanol-Induced TLR4/NLRP3 Neuroinflammatory Response in Microglial Cells Promotes Leukocyte Infiltration Across the BBB. Neurochem. Res..

[68] King J.A., Nephew B.C., Choudhury A., Poirier G.L., Lim A., Mandrekar P. (2020). Chronic alcohol-induced liver injury correlates with memory deficits: Role for neuroinflammation. Alcohol.

[69] Huf F., Bandiera S., Muller C.B., Gea L., Carvalho F.B., Rahmeier F.L., Reiter K.C., Tortorelli L.S., Gomez R., da Cruz Fernandes M. (2019). Comparative study on the effects of cigarette smoke exposure, ethanol consumption and association: Behavioral parameters, apoptosis, glial fibrillary acid protein and S100beta immunoreactivity in different regions of the rat hippocampus. Alcohol.

[70] Gessa G.L., Muntoni F., Collu M., Vargiu L., Mereu G. (1985). Low doses of ethanol activate dopaminergic neurons in the ventral tegmental area. Brain Res..

[71] Brodie M.S., Shefner S.A., Dunwiddie T.V. (1990). Ethanol increases the firing rate of dopamine neurons of the rat ventral tegmental area in vitro. Brain Res..

[72] Yan Q.S., Reith M.E., Jobe P.C., Dailey J.W. (1996). Focal ethanol elevates extracellular dopamine and serotonin concentrations in the rat ventral tegmental area. Eur. J. Pharmacol..

[73] Ortiz J., Fitzgerald L.W., Charlton M., Lane S., Trevisan L., Guitart X., Shoemaker W., Duman R.S., Nestler E.J. (1995). Biochemical actions of chronic ethanol exposure in the mesolimbic dopamine system. Synapse.

[74] Van Eldik L.J., Wainwright M.S. (2003). The Janus face of glial-derived S100B: Beneficial and detrimental functions in the brain. Restor. Neurol. Neurosci..

[75] Ben Haim L., Carrillo-de Sauvage M.A., Ceyzeriat K., Escartin C. (2015). Elusive roles for reactive astrocytes in neurodegenerative diseases. Front. Cell Neurosci..

[76] Zakiniaeiz Y., Scheinost D., Seo D., Sinha R., Constable R.T. (2017). Cingulate cortex functional connectivity predicts future relapse in alcohol dependent individuals. Neuroimage Clin..

[77] Bauer J., Pedersen A., Scherbaum N., Bening J., Patschke J., Kugel H., Heindel W., Arolt V., Ohrmann P. (2013). Craving in alcohol-dependent patients after detoxification is related to glutamatergic dysfunction in the nucleus accumbens and the anterior cingulate cortex. Neuropsychopharmacology.

[78] Rowniak M., Bogus-Nowakowska K., Robak A. (2015). The densities of calbindin and parvalbumin, but not calretinin neurons, are sexually dimorphic in the amygdala of the guinea pig. Brain Res..

[79] Price M.E., McCool B.A. (2022). Structural, functional, and behavioral significance of sex and gonadal hormones in the basolateral amygdala: A review of preclinical literature. Alcohol.

[80] Baker A.E., Brautigam V.M., Watters J.J. (2004). Estrogen modulates microglial inflammatory mediator production via interactions with estrogen receptor beta. Endocrinology.

[81] Barreto G., Veiga S., Azcoitia I., Garcia-Segura L.M., Garcia-Ovejero D. (2007). Testosterone decreases reactive astroglia and reactive microglia after brain injury in male rats: Role of its metabolites, oestradiol and dihydrotestosterone. Eur. J. Neurosci..

[82] Yang L., Tong Y., Chen P.F., Miao S., Zhou R.Y. (2020). Neuroprotection of dihydrotestosterone via suppression of the toll-like receptor 4/nuclear factor-kappa B signaling pathway in high glucose-induced BV-2 microglia inflammatory responses. Neuroreport.

[83] Liu X., Fan X.L., Zhao Y., Luo G.R., Li X.P., Li R., Le W.D. (2005). Estrogen provides neuroprotection against activated microglia-induced dopaminergic neuronal injury through both estrogen receptor-alpha and estrogen receptor-beta in microglia. J. Neurosci. Res..

[84] Mineur Y.S., Garcia-Rivas V., Thomas M.A., Soares A.R., McKee S.A., Picciotto M.R. (2022). Sex differences in stress-induced alcohol intake: A review of preclinical studies focused on amygdala and inflammatory pathways. Psychopharmacology.

